# Improving retention in antenatal and postnatal care: a systematic review of evidence to inform strategies for adolescents and young women living with HIV

**DOI:** 10.1002/jia2.25770

**Published:** 2021-08-27

**Authors:** Kirsty Brittain, Chloe A Teasdale, Bernadette Ngeno, Judith Odondi, Boniface Ochanda, Karryn Brown, Agnes Langat, Surbhi Modi, Elaine J Abrams

**Affiliations:** ^1^ Division of Epidemiology & Biostatistics School of Public Health & Family Medicine University of Cape Town Cape Town South Africa; ^2^ Centre for Infectious Disease Epidemiology & Research School of Public Health & Family Medicine University of Cape Town Cape Town South Africa; ^3^ Mailman School of Public Health ICAP‐Columbia University New York NY USA; ^4^ Department of Epidemiology Mailman School of Public Health New York NY USA; ^5^ Department of Epidemiology and Biostatistics CUNY Graduate School of Public Health & Health Policy New York NY USA; ^6^ Division of Global HIV and Tuberculosis (DGHT) US Centers for Disease Control and Prevention Atlanta GA USA; ^7^ Division of Global HIV and Tuberculosis (DGHT) US Centers for Disease Control and Prevention Nairobi Kenya; ^8^ Department of Pediatrics Vagelos College of Physicians and Surgeons Columbia University New York NY USA

**Keywords:** prevention of mother‐to‐child transmission, adolescents living with HIV, retention, adolescent pregnancy, antenatal care, HIV cascade

## Abstract

**Introduction:**

Young pregnant and postpartum women living with HIV (WLHIV) are at high risk of poor outcomes in prevention of mother‐to‐child transmission services. The aim of this systematic review was to collate evidence on strategies to improve retention in antenatal and/or postpartum care in this population. We also conducted a secondary review of strategies to increase attendance at antenatal care (ANC) and/or facility delivery among pregnant adolescents, regardless of HIV status, to identify approaches that could be adapted for adolescents and young WLHIV.

**Methods:**

Selected databases were searched on 1 December 2020, for studies published between January 2006 and November 2020, with screening and data abstraction by two independent reviewers. We identified papers that reported age‐disaggregated results for adolescents and young WLHIV aged <25 years at the full‐text review stage. For the secondary search, we included studies among female adolescents aged 10 to 19 years.

**Results and discussion:**

Of 37 papers examining approaches to increase retention among pregnant and postpartum WLHIV, only two reported age‐disaggregated results: one showed that integrated care during the postpartum period increased retention in HIV care among women aged 18 to 24 years; and another showed that a lay counsellor‐led combination intervention did not reduce attrition among women aged 16 to 24 years; one further study noted that age did not modify the effectiveness of a combination intervention. Mobile health technologies, enhanced support, active follow‐up and tracing and integrated services were commonly examined as standalone interventions or as part of combination approaches, with mixed evidence for each strategy. Of 10 papers identified in the secondary search, adolescent‐focused services and continuity of care with the same provider appeared to be effective in improving attendance at ANC and/or facility delivery, while home visits and group ANC had mixed results.

**Conclusions:**

This review highlights the lack of evidence regarding effective strategies to improve retention in antenatal and/or postpartum care among adolescents and young WLHIV specifically, as well as a distinct lack of age‐disaggregated results in studies examining retention interventions for pregnant WLHIV of all ages. Identifying and prioritizing approaches to improve retention of adolescents and young WLHIV are critical for improving maternal and child health.

## 1 Introduction

Access to and uptake of antiretroviral therapy (ART) have improved the health of pregnant women living with HIV (WLHIV) and decreased new paediatric HIV infections [1, 2, 3], but retaining women in HIV services, particularly during the postpartum period, remains a global challenge [4, 5, 6]. Adolescents and youth aged <25 years are at high risk of disengagement from HIV care generally [7], and young pregnant and postpartum WLHIV are at particularly high risk of poor outcomes in prevention of mother‐to‐child transmission (PMTCT) services [8, 9, 10]. Compared to adult WLHIV in sub‐Saharan Africa, poor engagement in antenatal care (ANC), decreased uptake of and retention in PMTCT services, lower rates of infant HIV testing and higher rates of mother‐to‐child transmission have been observed in younger women [8, 9, 10]. Pregnant adolescents and young women without HIV also have poor health outcomes compared to adult mothers, including late entry into ANC and higher risks of pre‐term delivery, infants with low birthweight and maternal and infant mortality [11, 12, 13].

Reasons for these poor outcomes are likely multifactorial [14], and combination approaches may be needed to address the multiple overlapping risks that this group faces. Adolescence is a critical stage of biological and psychosocial development, and this transition period is further complicated by HIV and pregnancy [15]. Qualitative data suggest that barriers to care among adolescents and young women include stigma surrounding adolescent pregnancy and HIV infection, lack of social support, concerns about confidentiality and negative relationships with healthcare providers [14, 16], highlighting the importance of addressing the multiple needs of this vulnerable group. With approximately 30% of new HIV infections in sub‐Saharan Africa occurring in women younger than 25 years [17] and high rates of pregnancy during adolescence in this region [18], young pregnant and postpartum WLHIV are a priority population. However, standard PMTCT services have not been designed to address the unique needs of adolescents and young women [14]. For this vulnerable population to achieve optimal maternal and child health outcomes, evidence‐based strategies are needed, including new models for differentiated service delivery [19].

The aim of this systematic review was to collate the available evidence on strategies to improve retention in antenatal and/or postpartum care among adolescents and young WLHIV. We also conducted a secondary review of strategies to increase attendance at ANC and/or facility delivery among pregnant adolescents, regardless of HIV status. The purpose of this secondary review was to identify approaches that have been effective in the general adolescent population and could be adapted for pregnant and postpartum adolescents and young WLHIV. Given that WLHIV are at increased risk of poor retention during pregnancy and the postpartum period in particular [4, 5, 6], we did not consider interventions tested among non‐pregnant women.

## 2 Methods

This review was conducted following the Preferred Reporting Items for Systematic Reviews and Meta‐Analyses (PRISMA) guidelines [20]. Search methods were discussed and finalized before beginning the searches, and the two searches were conducted on December 1, 2020. Searches were conducted in PubMed and Scopus and were restricted to English language articles published between January 2006 (corresponding with the date when lifelong ART was first recommended for pregnant and breastfeeding women based on disease staging [21]) and November 2020. No restrictions were placed on study design or geographical location for either search. The search terms were selected based on similar systematic reviews [22, 23, 24, 25] and are presented in Table S1. Two of these reviews focused on pregnant women of all ages [22, 23], and the others focused on non‐pregnant adolescents and youth living with HIV [24, 25]. Titles and abstracts of retrieved studies were merged and de‐duplicated in Mendeley. Two independent reviewers screened titles and abstracts for relevance, followed by full‐text review of all potentially relevant articles. Conference abstracts were not searched; letters, editorials, review articles and commentaries were excluded; and we did not contact authors for additional study details except for studies that included authors of this review in which case additional data were provided (where noted). Reference lists of all included studies and relevant review articles were screened for additional references. For both searches, data from eligible studies were extracted into a standardized Microsoft Excel table by the two independent reviewers.

We defined adolescents as women aged 10 to 19 years and young women as those aged 20 to 24 years. For the primary search of strategies to improve retention in pregnant adolescents and young WLHIV, the following criteria were used to screen abstracts: (1) study population included pregnant and/or postpartum WLHIV; (2) study population included women aged <25 years; (3) study examined an approach to increase maternal retention in antenatal and/or postpartum care and (4) study included a comparison group. Although our aim was to review strategies to improve retention specifically among pregnant adolescents and young WLHIV, we reviewed full texts of all studies with any pregnant participants aged <25 years, including those with participants aged ≥25 years. This was done to identify all effective approaches to retaining WLHIV in antenatal and postnatal services, even those that have not been specifically targeted to younger women, as these approaches could potentially be adapted for younger women specifically. At the full‐text review stage, we identified papers that reported age‐disaggregated results (i.e. effective approaches for women aged <25 years), and we present results separately for papers with and without age‐disaggregated results. Interventions that appear to be effective overall may not improve retention among younger women specifically, and identifying differences by age is critical to refine interventions for this vulnerable population.

For the secondary search of approaches aimed at retaining pregnant adolescents regardless of HIV status, the following criteria were used: (1) study population included pregnant adolescents; (2) study examined an approach to increase attendance at ANC and/or facility delivery; and (3) study included a comparison group. No restrictions were placed on study design or location. For this search, search terms such as “adolescent,” “teenager” and “youth” were used to limit the retrieved studies to adolescent populations, thereby identifying approaches that were examined specifically for adolescents. As above, we defined adolescents as women aged 10 to 19 years but included studies with women aged up to 22 years if authors identified these women as part of the adolescent population. Unlike the primary search, the secondary search was limited to studies examining outcomes among only adolescents as they are considered a high‐risk population for poor health outcomes, whereas younger pregnant women (>20 years) have not been a focus.

## 3 Results and discussion

### 3.1 Primary search: strategies to improve retention in care among adolescents and young WLHIV

**Figure 1:**
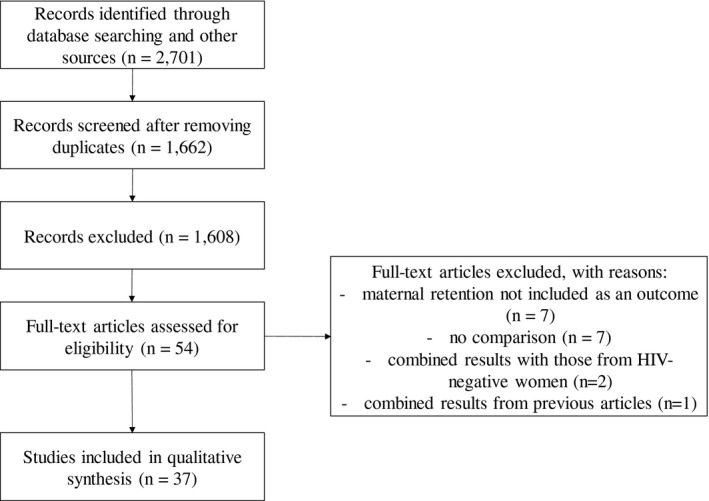
Preferred reporting items for systematic reviews and meta‐analyses (PRISMA) flow diagram summarising the primary search of approaches to improve retention in antenatal and/or postpartum care among adolescents and young women living with HIV.

The initial search yielded 2701 abstracts (Figure 1). After removing duplicates (n = 1039) and non‐relevant titles and abstracts (n = 1608), 54 full‐text articles were assessed for eligibility. Of these, 17 were excluded for not evaluating maternal retention as an outcome (n = 7), not including a comparison group (n = 7), including HIV‐negative women and not reporting results separately for WLHIV (n = 2), or combining the results of previously reported studies (n = 1). We found 37 papers examining an approach to increase maternal retention in antenatal and/or postpartum care in samples that included adolescent and young WLHIV. Notably, only two papers presented age‐disaggregated results [26, 27] and one additional paper noted that age did not modify the study results [28]. Among all 37 papers evaluating at least one approach to increase retention, 18 demonstrated no effect on retention [29, 30, 31, 32, 33, 34, 35, 36, 37, 38, 39, 40, 41, 42, 43, 44, 45, 46] and 19 reported a significant effect on retention [26, 27, 28, 47, 48, 49, 50, 51, 52, 53, 54, 55, 56, 57, 58, 59, 60, 61, 62]. Notably, several papers reported significant improvements in retention at some time points but not at others; these are categorized as reporting a significant effect on retention. The results of two intervention studies are reported in two papers, respectively [26, 38, 39, 40], but in each case, both papers are included as they report different retention outcomes.

All 37 papers included in the review reported results from studies conducted in sub‐Saharan Africa, including eight papers from South Africa [26, 30, 38, 39, 40, 42, 46, 48]. One study was published during 2010 [30], but the remainder were published between 2014 and 2020. Cluster‐randomized controlled trials were the most common study design [29, 32, 34, 35, 36, 37, 39, 40, 43, 47, 56, 57, 58], and the duration of follow‐up across studies ranged from one month [28, 30, 33, 61] to 36 to 60 months’ postpartum [38], with two‐thirds of studies following women through ≤6 months’ postpartum [27, 28, 30, 31, 33, 35, 36, 37, 39, 40, 41, 43, 44, 45, 47, 48, 50, 53, 54, 55, 56, 59, 60, 61, 62]. Many studies were restricted to women who were not yet receiving ART when presenting for ANC or delivery, and 12 studies were restricted to women who were aged ≥18 years at enrolment in the study [26, 28, 29, 33, 36, 41, 43, 45, 46, 48, 55, 61].

### 3.2 Studies presenting results among adolescents and young WLHIV specifically

The two studies that presented age‐disaggregated results [26, 27] and one that noted that age did not modify the study results [28] allow an examination of the effectiveness of these interventions among adolescents and young WLHIV specifically (Table 1). The MCH‐ART study, conducted in South Africa, examined the effectiveness of integrated care during the postpartum period, defined as the co‐delivery of services at the same time and location. This randomized controlled trial was conducted among 471 women (mean age, 29 years) who initiated ART during pregnancy and opted to breastfeed [26]. During pregnancy, all women received integrated antenatal and HIV care within the Midwife Obstetric Unit, consistent with the local standard of care. Women were randomized immediately postpartum to either continued integrated care within the Midwife Obstetric Unit through the end of breastfeeding, including maternal ART care and routine infant care at the same visits, or the local standard of postpartum care (referral out to general adult ART services, with infant care provided at separate child health clinics). Women in the intervention arm were referred out to general adult ART services at a median of 32 weeks' postpartum, compared to 11 days in the control arm. The primary outcome was a composite endpoint of maternal retention in routine HIV care at 12 months’ postpartum and viral suppression <50 copies/mL. Women in the intervention arm were significantly more likely to be retained in care and virally suppressed at 12 months’ postpartum (77% vs. 56% in the standard of care arm; *p* < 0.001) [26]. Compared to women aged ≥25 years, younger women were less likely to be retained in care and virally suppressed in both the intervention (61% vs. 82%) and the control arm (41% vs. 62%; MCH‐ART study team, personal communication). In addition, the integrated care intervention effectively improved retention in care and viral suppression at 12 months’ postpartum among women aged <25 years at enrolment (61% among women in the intervention arm vs. 41% among those in the control arm; MCH‐ART study team, personal communication). Following completion of the MCH‐ART trial, follow‐up was extended to 36 to 60 months’ postpartum [38]. Retention data were available for 450 women (96% of the original cohort), with no difference in retention in care by original allocation (63% of women in both arms; *p* = 0.885) and loss from care occurring soon after transfer out of the integrated clinic [38].

**Table 1:** Studies presenting results of approaches to improve retention in antenatal and/or postpartum care among adolescents and young women living with HIV specifically

Author; country; years	Journal; year of publication	Study design and intervention	Eligibility, including age	Sample characteristics	Maternal retention outcome(s) among women of all ages	Outcome(s) among adolescents and young women specifically
Fayorsey; Kenya; 2013 to 2014 [27]	J Acquir Immune Defic Syndr; 2019	RCT: Lay counsellors provided (1) individualized PMTCT education; (2) retention and adherence support; (3) phone and SMS appointment reminders; (4) tracking for missed visits	Pregnant women aged ≥16 years and with access to a cell phone	Intervention: n = 170, 37% aged <25 years. Control: n = 170, 45% aged <25 years	Lower levels of attrition among mother–infant pairs in intervention at six months’ postpartum (19% in intervention vs. 28%)	No difference in attrition among mother–infant pairs at six months’ postpartum (31% in intervention vs. 32%)
Mubiana‐Mbewe; Zambia; 2017 [28]	AIDS Behav; 2020	RCT: Community workers provided follow‐up for missed visits and optional services, including individual counselling, home‐based cHTC, male partner HIV testing and appointment reminders	Pregnant women aged ≥18 years with no previous ART use or current ART use for <7 days	Intervention: n = 229, median age 26 years. Control: n = 225, median age 28 years	No difference in retention in care after 30 days in intention‐to‐treat analysis. In per‐protocol analysis, intervention associated with higher levels of retention (92% in intervention vs. 80%)	Authors noted that age did not modify the intervention effect (no further details provided)
Myer; South Africa; 2013 to 2014 [26]	PLoS Med; 2018	RCT: Integrated postnatal care for mothers and infants through the end of breastfeeding	RCT: Breastfeeding women aged ≥18 years who had initiated ART during pregnancy and were <6 weeks’ postpartum	Intervention: n = 233, mean age, 29 years. Control: n = 238, mean age, 29 years	Intervention associated with higher proportion of mothers retained in care and virally suppressed at 12 months’ postpartum (77% in intervention vs. 56%)	Intervention associated with higher proportion of young mothers retained in care and virally suppressed at 12 months’ postpartum (61% in intervention vs. 41%)

ART, antiretroviral therapy; cHTC, couple HIV testing and counselling; PMTCT, prevention of mother‐to‐child transmission; RCT, randomized controlled trial; SMS, short message service.

The second study, MIR4Health, was a randomized controlled trial of a lay counsellor‐led combination intervention conducted among 340 Kenyan women who were aged 16 years or older (median age, 26 years; 41% <25 years of age) and had access to a cell phone [27]. Women randomized to the intervention were assigned a lay counsellor who provided individualized PMTCT health education, support for retention and adherence, telephonic and text message appointment reminders, and follow‐up and tracking for missed clinic visits. The primary outcome was mother–infant attrition at six months’ postpartum due to maternal or infant death or loss to follow‐up. In the intervention arm, attrition of mother–infant pairs at six months’ postpartum was significantly lower than in the standard of care arm (19% vs. 28% in the standard of care arm; *p* = 0.04). However, the intervention effect was modified by maternal age, with no significant difference in attrition across arms among women aged 16 to 24 years at enrolment (31% vs. 32% in the standard of care arm; *p* = 0.96) [27].

Finally, a randomized controlled trial conducted in Zambia examined the effect of an Option B+ Enhanced Adherence Package (BEAP) on initiation of ART and retention at 30 days [28]. The BEAP used community workers to provide follow‐up for missed visits as well as other optional services, including individual counselling, home‐based couples counselling and testing, male partner HIV testing and appointment reminders. The study enrolled 454 pregnant WLHIV aged 18 years or older (median age, 27 years) who had no previous ART use or had initiated ART during the past seven days. This study found no effect on retention after 30 days in an intention‐to‐treat analysis, although the intervention was found to significantly improve retention in a per‐protocol analysis (92% vs. 80% in the control arm). The authors noted that age did not modify the intervention effect but did not state the age groups examined in these sub‐group analyses [28].

### 3.3 Learning from strategies to improve retention among adolescents and young WLHIV

Despite repeated calls for the development of evidence‐based approaches to improve retention in antenatal and/or postpartum care among adolescents and young WLHIV, this review highlights the lack of evidence regarding effective strategies for these groups. We identified only two papers that reported results separately for adolescents and young WLHIV, and one that noted that age did not modify the intervention effect. Two of the three studies were restricted to women aged 18 years or older at enrolment [26, 28]. The MIR4Health combination intervention study improved mother‐infant retention at six months’ postpartum in the overall sample, but not among those aged 16 to 24 years at enrolment [27]. In contrast, age did not modify the effect of the BEAP combination intervention on retention after 30 days [28]. The MCH‐ART study examined the effectiveness of integrated postpartum care, and showed that this intervention is effective in improving retention through 12 months’ postpartum among women of all ages as well as women aged 18 to 24 years specifically [26]. This comparatively simple intervention may be effective through a range of mechanisms, including a lower burden of clinic visits as well as less HIV‐related stigma through receiving ART in the Midwife Obstetric Unit. For young women who may receive limited financial support and have specific concerns related to stigma, these mechanisms may be particularly important. In addition, the MCH‐ART intervention has been shown to be cost‐effective [63], which is of particular importance in resource‐limited settings, and we believe that this approach warrants further exploration.

### 3.4 Studies examining interventions among WLHIV of all ages

The remaining 34 papers examined the effectiveness of interventions among WLHIV of all ages (Table 2). Although this does not allow conclusions to be drawn about the effectiveness of these interventions among adolescents and young women, the effective interventions identified could be examined among younger women specifically or adapted where necessary. The most frequently examined approaches in these studies were the use of mobile health (mHealth) technologies, often in the form of SMS messaging; enhanced support for mothers, typically in the form of additional social support provided to individual mothers by peer or mentor supporters or during group counselling; active follow‐up and tracing; and integrated PMTCT and ANC services (ART given in ANC clinics during pregnancy and sometimes postpartum) or integrated mother–infant care. As stated above, integrated care is defined as the co‐delivery of services at the same time and location. These approaches were either examined as standalone interventions or as part of combination approaches, and we present results according to this distinction below.

**Table 2:** Studies examining approaches to improve retention in antenatal and/or postpartum care among women living with HIV of all ages

Author; country; years	Journal; year of publication	Study design and intervention	Eligibility, including age	Sample characteristics	Maternal retention outcome(s)
Papers that reported that the approach examined did not significantly improve maternal retention					
Foster; Zimbabwe; 2014 to 2015 [29]	J Acquir Immune Defic Syndr; 2017	Cluster RCT: Mother support groups from entry into ANC through six months’ postpartum	Pregnant women aged ≥18 years and <35 weeks of gestation	Intervention: n = 188, 34% aged 18 to 27 years. Control: n = 160, 41% aged 18 to 27 years	No difference in attendance at 12 months’ postpartum between study arms (71% in intervention vs. 61%) or regular attendance through 12 months’ postpartum (78% in intervention vs. 71%)
Futterman; South Africa; 2006 to 2007 [30]	AIDS Care; 2010	Pilot comparing intervention clinic to control clinic: Two‐part intervention: (1) mentor mothers provided support through pregnancy and after delivery; and (2) 8‐session group cognitive‐behavioural intervention	Pregnant women diagnosed HIV+ during ANC; no restrictions based on age specified	Intervention: n = 83, mean age, 26 years. Control: n = 77, mean age, 27 years	No difference in self‐reported attendance at a post‐delivery follow‐up visit (58% in intervention vs. 36%)
Gamell; Tanzania; 2008 to 2014 [31]	J Acquir Immune Defic Syndr; 2016	Before‐after cohort study: Intervention package: (1) integrated PMTCT and MCH services; (2) electronic medical records; (3) provider‐initiated HTC in hospital wards and (4) EID tests locally	Pregnant women; no restrictions based on age specified	Before intervention: n = 110, mean age, 30 years. During/after intervention: n = 90, mean age, 30 years	No differences in retention in care six months after enrolment across study periods (62% both before and during/after)
Joseph; Zimbabwe; 2014 to 2015 [32]	J Acquir Immune Defic Syndr; 2017	Cluster RCT: Point‐of‐care CD4 testing with CD4 count‐specific adherence counselling	ART‐naïve pregnant women entering ANC; no restrictions based on age specified	Intervention: n = 603, median age, 26 years. Control: n = 547, median age, 27 years	No difference in retention after six months (59% in intervention vs. 63%) or 12 months (51% in intervention vs. 54%) on ART
Kim; Malawi; 2016 to 2018 [33]	AIDS Behav; 2019	Pilot RCT: ART educational video followed by a question and answer session with a healthcare worker prior to ART initiation	Pregnant women not on ART at entry into ANC, aged ≥18 years or ≥16 years and either married or having given birth to a previous child	Intervention: n = 146, mean age 28 years. Control: n = 160, mean age 27 years	No difference in retention after one month (77% in intervention vs. 75%)
Mwapasa; Malawi; 2013 to 2016 [34]	J Acquir Immune Defic Syndr; 2017	Cluster RCT: Compared 2 interventions to control: (1) Integrated HIV and MNCH services; and (2) integrated services and SMS reminders	Pregnant women on ART; no restrictions based on age specified	Intervention 1: n = 461, median age, 30 years. Intervention 2: n = 493, median age, 29 years. Control: n = 396, median age, 29 years	No difference in proportion of mothers retained in care through 12 months’ postpartum (in intervention (1) 19%; intervention (2) 25%; control 22%)
Nance; Tanzania; 2015 to 2016 [35]	PLoS One; 2017	Cluster RCT: Intervention package: (1) Mentorship and supervision of CHWs; (2) birth planning cards; (3) adherence counselling by CHWs; (4) defaulter tracing by CHWs	Postpartum women; no restrictions based on age specified	Intervention: n = 909. Control: n = 1044. Age not reported	No difference in proportion of mothers retained in care at 90 days postpartum (45% in intervention vs. 47%)
Odeny; Kenya; 2015 to 2016 [36]	PLoS Med; 2019	Stepped‐wedge cluster RCT: Text messages during pregnancy and after delivery, with the option of responding to messages or calling a nurse	Pregnant women aged ≥18 years or emancipated minors and ≥28 weeks’ gestation	Intervention: n = 1764, 34% aged <25 years. Control: n = 751, 37% aged <25 years	No difference in retention at eight weeks’ postpartum (90% in intervention vs. 76%)
Oyeledun; Nigeria; 2014 to 2016 [37]	J Acquir Immune Defic Syndr; 2017	Cluster RCT: Continuous quality improvement, including improvements in time spent at clinic; attitudes of health workers; client satisfaction; provision of ART at ANC visits	Pregnant women <34 weeks’ gestation and initiating ART; no restrictions based on age specified	Intervention: n = 264, 34% aged <25 years. Control: n = 247, 35% aged <25 years	No difference in retention in care through six months (44% in intervention vs. 41%)
Phillips; South Africa; 2017 to 2018 [38]	J Acquir Immune Defic Syndr; 2020	RCT: Integrated postnatal care for mothers and infants through the end of breastfeeding (long‐term follow‐up of Myer *et al*., 2018, below)	RCT: Breastfeeding women aged ≥18 years who had initiated ART during pregnancy and were <6 weeks’ postpartum	Intervention: n = 223. Control: n = 227	No difference in retention in care at 36 to 60 months’ postpartum (63% in intervention vs. 63%)
Richter; South Africa; 2008 to 2010 [39]	AIDS Behav; 2014	Cluster RCT: Four antenatal and four postnatal small group sessions led by peer mentors	Pregnant women at their first ANC visit; no restrictions based on age specified	Intervention: n = 544, mean age, 27 years. Control: n = 656, mean age, 27 years	No difference in proportion attending 4+ ANC visits across arms (87% in intervention vs. 76%)
Rotheram‐Borus; South Africa; 2008 to 2010 [40]	PLoS One; 2014	This paper reports different outcomes from the study above, Richter *et al*., 2014	As above	As above	No difference in proportion attending at least one postpartum visit across arms (63% in intervention vs. 45%)
Sabin; Uganda; 2015 to 2016 [41]	AIDS Behav; 2020	RCT: Use of Wisepill wireless pill monitors with text message reminders triggered by late dose‐taking, and data‐informed counselling at clinic visits	Pregnant women aged ≥18 years, between 12 and 26 weeks’ gestation, initiating ART and with access to a cellphone	Intervention: n = 69, mean age 26 years. Control: n = 64, mean age 25 years	No difference in any of several retention measures through three months’ postpartum
Schwartz; South Africa; 2013 [42]	Matern Child Health J; 2015	Pilot compared to a retrospective cohort: weekly text messages from a case manager through six weeks’ postpartum, and one pre‐delivery and two post‐delivery telephone calls. Participants able to send “Please Call Me” text messages for additional phone calls	Pregnant women ≥36 weeks’ gestation who own a cell phone and can read text messages in English; no restrictions based on age specified	Intervention: n = 50, median age, 28 years. Control: n = 50, median age, 29 years	No difference in proportion of women engaged in care at 10 weeks (94% in intervention vs. 96%) or proportion lost to follow‐up by 12 months (22% in intervention vs. 24%)
Turan; Kenya; 2009 to 2011 [43]	J Acquir Immune Defic Syndr; 2015	Cluster RCT: Integrated HIV and PMTCT services through 18 months’ postpartum	Pregnant women aged ≥18 years who were not enrolled in HIV care at baseline	Intervention: n = 569, mean age, 25 years. Control: n = 603, mean age, 25 years	No difference in proportion attending 2+ HIV care visits in first six months after entering care (48% in intervention vs. 56%)
Vogt; Zimbabwe; 2010 to 2013 [44]	J Int AIDS Soc; 2015	Before‐after intervention study: CHW‐based defaulter tracing system to find women lost from care	Pregnant women newly enrolled into PMTCT; no restrictions based on age specified	Before: n = 1278, 35% aged <25 years. After: n = 600, 38% aged <25 years	No difference in retention at delivery between arms (84% before intervention vs. 86% after)
Watt; Tanzania; 2019 [45]	AIDS Behav; 2020	Pilot RCT: HIV stigma counselling intervention delivered at up to three sessions after entry into antenatal care	Pregnant women aged ≥18 years, living with HIV and entering antenatal care. Intervention also targeted women living without HIV	Intervention: n = 28. Control: n = 27. Age data combined with data from women living without HIV	No difference in retention after three months of follow‐up (89% in both intervention and control)
Zerbe; South Africa; 2015 [46]	BMC Health Serv Res; 2020	Cohort study: Postpartum women given the choice of attending community‐based adherence clubs or primary care clinics for HIV services	Recently postpartum women aged ≥18 years who had initiated ART during their recent pregnancy, were currently breastfeeding, and had viral load <1000 copies/mL after 12 weeks on ART	Adherence clubs: n = 84, 26% aged <25 years. Primary care clinics: n = 45, 29% aged <25 years	No difference in retention in care at 12 months’ postpartum (88% among women choosing adherence clubs vs. 82% among women choosing primary care clinics)
Papers that reported that the approach examined significantly improved maternal retention					
Aliyu; Nigeria; 2013 to 2014 [47]	Lancet HIV; 2016	Cluster RCT: Package including point‐of‐care CD4 testing; task shifting; integrated postpartum MCH services; male partner invitation letters; male‐friendly health services; community mobilization	Women presenting for ANC or delivery and not on ART or ARVs; no restrictions based on age specified	Intervention: n = 172, median age, 26 years. Control: n = 197, median age, 28 years	Increased retention at six weeks’ postpartum (83% in intervention vs. 9%) and at 12 weeks postpartum (75% in intervention vs. 7%)
Coleman; South Africa; 2013 to 2014 [48]	AIDS Care; 2017	Retrospective intervention study: Maternal health‐related SMS sent throughout pregnancy and through 12 months’ postpartum	Pregnant women aged ≥18 years and with access to a cell phone	Intervention: n = 235, 12% aged <23 years. control: n = 586, 13% aged <23 years	Intervention group attended more ANC visits (average of 4.8 in intervention vs. 4.3, adjusted for timing of first ANC visit; 82% in intervention group attended 4+ ANC visits vs. 59% in control)
Flax; Uganda; 2011 to 2018 [49]	J Acquir Immune Defic Syndr; 2020	Retrospective cohort: Quality improvement approach, including integration of HIV and maternal and child health services; improvement of tracking and retention of mother‐infant pairs; staff training; mentor mother counselling; and improving data completeness. Approach first implemented in demonstration facilities, followed by scale‐up facilities	Facility‐level intervention	Demonstration facilities: n = 4517, median age 27 years. Scale‐up facilities: n = 1720, median age 29 years. Comparison facilities: n = 2787, median age 29 years	Retention in care at 12 months’ postpartum increased significantly in demonstration facilities; but no difference in retention at scale‐up versus comparison facilities
Herlihy; Zambia; 2011 to 2013 [50]	J Acquir Immune Defic Syndr; 2015	Pre‐post intervention study: Intervention included (1) integrated HIV and ANC services; (2) training of ANC providers and lay counsellors, including PMTCT and EID training; (3) expediting CD4 results; (4) follow‐up of mother‐infant pairs by lay counsellors	Pregnant women not on ART when entering ANC; no restrictions based on age specified	Pre‐intervention: n = 510, mean age, 26 years. Post‐intervention: n = 624, mean age, 27 years	After intervention, mothers attended more ANC visits (mean, 2.28 vs. 2.03); after intervention, fewer mothers were lost to follow‐up (15% vs. 25%)
Kinyua; Kenya; 2013 to 2016 [51]	J Int Assoc Provid AIDS Care; 2019	Retrospective cohort: Quality improvement approach, including improved documentation, integration of HIV and maternal and child health services and use of CHWs to assist in nutritional screening	Facility‐level intervention	Not reported at individual level	Retention in care improved consistently throughout the intervention period
Lerotholi; Lesotho; 2013 to 2016 [52]	J Int Assoc Provid AIDS Care; 2019	Retrospective cohort: Quality improvement approach (no further details reported in manuscript)	Facility‐level intervention	Not reported at individual level	Retention in care at 24 months’ postpartum improved significantly in the private hospital but not in the government hospital
Mwita; Tanzania; 2013 to 2015 [53]	J Int Assoc Provid AIDS Care; 2019	Retrospective cohort: Quality improvement approach, including integration of mother‐infant services, active follow‐up, improved documentation and use of CHWs to assist in nutritional screening	Facility‐level intervention	Not reported at individual level	Retention in care at postnatal visits improved throughout the intervention period
Namukwaya; Uganda; 2010 [54]	BMC Health Serv Res; 2015	Pre‐post intervention study: Counselling, home visits and community sensitization	Pregnant women; no restrictions based on age specified	Post‐intervention sample: n = 558 (median age, 26 years), compared to aggregate clinic data before intervention	Post‐intervention, higher levels of retention at six weeks’ postpartum (79% post‐intervention vs. 38%)
Odeny; Kenya; 2012 to 2013 [55]	AIDS; 2014	RCT: Two‐way, individually tailored SMS up until six weeks’ postpartum	Pregnant women aged ≥18 years and ≥28 weeks’ gestation who had access to a cell phone and could read SMS or had someone to read them	Intervention: n = 195, 31% aged 18 to 24 years. Control: n = 193, 34% aged 18 to 24 years	Higher proportion of mothers in intervention attended a clinic visit within eight weeks’ postpartum (20% in intervention vs. 12%)
Pfeiffer; Mozambique; 2014 to 2015 [56]	J Acquir Immune Defic Syndr; 2017	Stepped‐wedge cluster RCT: Enhanced retention package including workflow modifications, enhanced counselling and active patient tracking (texting, phone calls, home visits)	Women diagnosed HIV+ at first ANC visit and initiating ART within 14 days; no restrictions based on age specified	n = 761, approximately 60% aged <25 years	Intervention associated with higher levels of retention at 30 days (71% in intervention vs. 52%) and 60 days (58% in intervention vs. 46%) but not at 90 days (41% in intervention vs. 38%)
Phiri; Malawi; 2013 to 2014 [57]	J Acquir Immune Defic Syndr; 2017	3‐arm cluster RCT: Intervention (1): facility‐based peer support, with one‐on‐one support at each clinic visit, weekly support groups and follow‐ups for missed visits; intervention (2): community‐based peer support, including home visits, monthly support meetings and follow‐up for missed visits	ART‐naïve pregnant or breastfeeding women aged ≥15 years	Intervention (1): n = 428, 23% aged <22 years. Intervention (2): n = 394, 22% aged <22 years. Control: n = 447, 18% aged <22 years	No difference in retention at 12 months (intervention (1): 78%; intervention (2): 74%; control: 74%); retention at two years higher in both intervention arms (intervention (1): 80%; intervention (2): 83%; control: 66%)
Ross‐Degnan; Tanzania; 2014 to 2015 [58]	PLoS One; 2017	Group RCT: Appointment‐based and community‐based tracking systems in Option B+ clinics	Women on ART for ≥6 months who had attended 1+ clinic visit three to six months’ pre‐intervention; no restrictions based on age specified; note: not all women were currently pregnant	Intervention: n = 1924, 8% aged <20 years. Control: n = 1226, 5% aged <20 years	Rate of missed clinic visits decreased from 37% to 34% in intervention and increased from 39% to 46% in control
Sam‐Agudu; Nigeria; 2014 to 2015 [60]	J Acquir Immune Defic Syndr; 2017	Prospective paired cohort: Enhanced peer support from mentor mothers within a structured programme	Pregnant women; no restrictions based on age or gestation	Intervention: n = 260, 10% aged <21 years. Control: n = 237, 12% aged <21 years	Higher levels of retention at six months’ postpartum (62% in intervention vs. 25%)
Sarna; Kenya; 2013 to 2015 [59]	Glob Health Sci Pract; 2019	RCT: Individualized counselling delivered via cellphone, for a maximum of 42 sessions	Pregnant women aged ≥16 years, between 14 and 36 weeks of gestation and with access to a cellphone	Intervention: n = 207, median age 24 years. control: n = 197, median age 25 years	Higher levels of retention at delivery (95% in intervention vs. 78%); six weeks’ postpartum (94% vs. 73%); and 14 weeks’ postpartum (83% vs. 67%)
Wesevich; Malawi; 2014 [61]	AIDS Care; 2017	Secondary analysis of an RCT: Parent study: invitation letters and phone and physical tracing of male partners vs. invitation letters only to increase cHTC. This secondary analysis includes exploration of impact of cHTC	Pregnant women testing HIV+ through individual HTC at first ANC visit; aged ≥18 years or aged 16 to 17 years and married	Intervention: n = 126 control: n = 74. Median age overall, 26 years	Retention 30 days after ART initiation higher among those who received cHTC (87% vs. 65%)
Yotebieng; Democratic Republic of Congo; 2013 to 2014 [62]	J Acquir Immune Defic Syndr; 2016	RCT: Conditional cash transfers for attending clinic visits and accepting PMTCT services offered ($5, increasing by $1 at each subsequent visit)	Women newly diagnosed with HIV, ≤32 weeks’ gestation and registering for ANC; no restrictions based on age specified	Intervention: n = 216, median age, 30 years. Control: n = 217, median age, 29 years	Intervention associated with higher levels of retention in care at six weeks’ postpartum (81% in intervention vs. 72%)

ANC, antenatal care; ART, antiretroviral therapy; ARV, antiretroviral; cHTC, couple HIV testing and counselling; CHW, community health worker; EID, early infant diagnosis; HTC, HIV testing and counselling; MCH, maternal and child health; MNCH, maternal, neonatal and child health; PMTCT, prevention of mother‐to‐child transmission; RCT, randomized controlled trial; SMS, short message service.

Six studies investigated mHealth technologies as a standalone approach, with three investigating the use of text messages to improve retention. In South Africa, a study of one‐way, twice‐weekly maternal health information showed higher levels of attendance at ANC visits among women receiving text messages (82% of women in the intervention arm attended ≥4 ANC visits vs. 59% in the control arm) [48]. In Kenya, a randomized controlled trial of text messaging with the option of responding, calling or sending inquiry text messages showed higher levels of retention at 8 weeks’ postpartum (20% vs. 12% in the control) [55], while a larger cluster‐randomized controlled trial of the intervention showed no difference in retention at eight weeks’ postpartum [36]. A study in South Africa investigating both text messaging and telephone calls providing visit reminders, motivational support and health information showed no differences in retention at either 10 weeks' or 12 months’ postpartum [42]. In contrast, a study of individualized cellphone counselling in Kenya demonstrated higher levels of retention at each of delivery (95% vs. 78% in the control), six weeks' (94% vs. 73%) and 14 weeks’ postpartum (83% vs. 67%) [59]. Finally, a randomized controlled trial in Uganda used text message reminders triggered by late dose‐taking (monitored using Wisepill wireless pill monitors) but found no differences in retention through three months’ postpartum [41].

Five papers examined enhanced support for mothers as a standalone intervention, typically provided by mentor mothers or within groups, with four demonstrating no significant effect on retention [29, 30, 39, 40]. Two of these papers reported results from the same study of group‐based peer support in South Africa and showed no improvement in attendance at either antenatal [39] or postpartum care [40]. In contrast, a Nigerian study reported improved retention at six months’ postpartum related to an enhanced support intervention delivered by mentor mothers (62% in the intervention arm vs. 25% in the control arm) [60]. Two studies examined active follow‐up and tracing as a standalone intervention, with a before‐after intervention study of a defaulter tracing system showing no effect on retention at delivery in Zimbabwe [44], while a group‐randomized controlled trial of appointment‐ and community‐based tracking systems in Tanzania showed a decrease in missed clinic visits in the intervention arm (from 37% to 34%) and an increase in the control arm (from 39% to 46%) [58]. Finally, besides the MCH‐ART study and follow‐up study described above, a cluster‐randomized controlled trial in Kenya examined the effect of integrated services as a standalone intervention, but showed no effect of the intervention on retention during the first six months after entering care [43].

The remaining 12 studies examined combination interventions but did not report the effect of each component of the intervention. For those demonstrating effectiveness, it is thus not possible to identify the specific component(s) that improved retention. In addition, four of these studies used a continuous quality improvement approach, where the intervention was adapted and refined throughout the study period, which also does not allow for conclusions regarding the effectiveness of specific components. These included one study demonstrating no effect on retention in care through six months in Nigeria [37], two studies demonstrating improved retention in care in each of Kenya [51] and Tanzania [53] and one study from Uganda where retention in care at 12 months’ postpartum improved in demonstration but not at scale‐up facilities [49]. Three combination intervention studies showed no significant effect on retention. These included two studies from Tanzania, one of which tested an intervention package which included integrated services [31], and the other which tested an intervention package including adherence counselling and defaulter tracing by community health workers [35]. Finally, a 3‐arm cluster‐randomized controlled trial of (1) integrated services and (2) integrated services as well as SMS reminders, compared to the control, demonstrated no effect of either intervention on retention in care through 12 months’ postpartum in Malawi [34].

Alongside the MIR4Health, BEAP and continuous quality improvement studies described above, five other studies demonstrated the effectiveness of combination interventions, but it is not possible to identify the most effective intervention components. These components included integrated care [47, 50], active follow‐up and tracing [50, 54, 56, 57] and enhanced support through counselling [54, 56] or support groups [57]. The duration of follow‐up in most of these studies was short, ranging from increased retention during the antenatal period [50, 56] to increased retention at six weeks’ [54] and 12 weeks’ postpartum [47]; only one study reported outcomes through two years [57]. This 3‐arm cluster‐randomized controlled trial examined (1) facility‐based peer support and (2) community‐based peer support, with support including individual‐ and group‐based support as well as follow‐up for missed visits, versus the standard of care. No difference in retention was observed at 12 months after ART initiation, but retention after 24 months was significantly higher in both intervention arms compared to the control (80% and 83% vs. 66%) [57].

### 3.5 Learning from strategies to improve retention among WLHIV of all ages

Taken together, studies examining standalone interventions among WLHIV of all ages demonstrated mixed evidence for the effectiveness of these approaches on retention, including mHealth technologies (with three of six studies showing no effect on retention) and enhanced support provided by mentor mothers or within groups (with four of five studies showing no effect). One of two studies demonstrated the effectiveness of active follow‐up and tracing, and one study showed no effect of integrated services during the first six months after entering care, in contrast to the MCH‐ART study described above. The 12 studies examining combination interventions similarly showed mixed effectiveness and, as noted above, it is not possible to identify the effectiveness of individual components of these interventions.

Previous reviews have similarly shown mixed evidence for approaches to improve retention among WLHIV [22], including the effectiveness of integrated services [64, 65]. Although our review found mixed evidence for the effectiveness of active follow‐up and tracing, a previous review found that outreach services improved retention in PMTCT care among women of all ages [66]. In addition, peer support services and support groups have been found to improve retention in PMTCT care among women of all ages [66] and among men and WLHIV [67]. However, although peer support for adolescents living with HIV has been described as a promising approach to improve retention, there are few rigorous evaluations of this approach [68]. A recent cluster‐randomized controlled trial in Zimbabwe demonstrated higher levels of viral suppression among non‐pregnant adolescents who received a peer‐led community‐based support intervention (the Zvandiri intervention) compared to the standard of care [69], but evaluations among pregnant adolescents specifically are needed. Finally, it should be noted that interventions that are universally applied to WLHIV of all ages may result in less stigma compared to interventions for adolescents and young women specifically, but interventions that are effective overall should be examined in this vulnerable population.

### 3.6 Secondary search: strategies to improve retention among adolescents, regardless of HIV status

**Figure 2:**
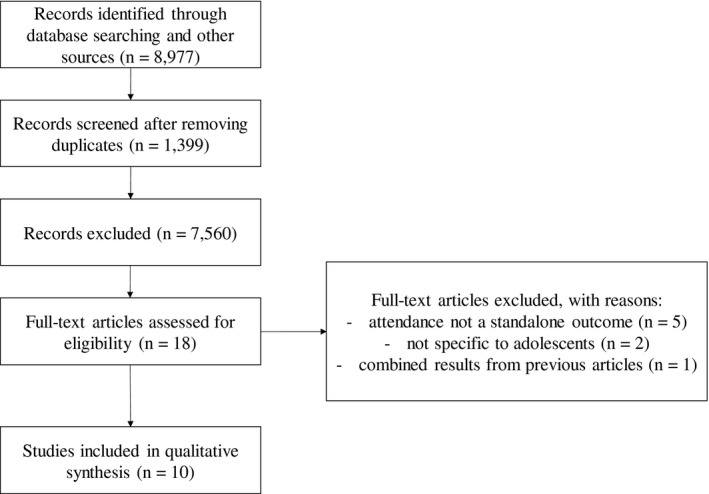
Preferred reporting items for systematic reviews and meta‐analyses (PRISMA) flow diagram summarising the secondary search of approaches to improve attendance at antenatal care and/or facility delivery among adolescents, regardless of HIV status.

This secondary search yielded 8977 abstracts (Figure 2). After removing duplicates (n = 1399) and non‐relevant titles and abstracts (n = 7560), 18 full‐text articles were assessed for eligibility. Of these, eight were excluded for not evaluating attendance as a standalone outcome (n = 5), not targeting adolescents specifically (n = 2), and for reporting combined results from previously published papers (n = 1), resulting in 10 papers that examined approaches to increase attendance at ANC and/or facility delivery among adolescents (Table 3). Two major differences between the results of the two searches are evident. In contrast to the primary search, most of the 10 studies identified in the secondary search were conducted in high‐income countries, including five in the United States [70, 71, 72, 73, 74]. In addition, cluster‐randomized controlled trials were the most common study design identified in the primary search, but only three of the 10 studies identified in the secondary search randomized participants [72, 75, 76]. Seven of the studies reported that the intervention was associated with improved attendance at ANC appointments [70, 71, 74, 75, 77, 78, 79]; only one study examined facility delivery as a standalone outcome but the intervention was not effective in improving this outcome [79]. Although these studies do not allow conclusions to be drawn about the effectiveness of these interventions among adolescents and young WLHIV, the effective interventions identified could be examined among WLHIV specifically or adapted where necessary.

**Table 3:** Studies examining approaches to improve attendance at antenatal care and/or facility delivery among adolescents, regardless of HIV status

Author; country; years	Journal; year of publication	Study design and intervention	Eligibility, including age	Sample characteristics	ANC attendance outcome(s)
Papers that reported that the approach examined did not significantly improve ANC attendance					
Ickovics; USA; 2008 to 2012 [72]	Am J Public Health; 2016	Cluster RCT: Centering pregnancy with reproductive health promotion	Adolescents aged 14 to 21 years; entering ANC at <24 weeks’ gestation; not high‐risk pregnancy; able to speak English or Spanish	Intervention: n = 610, mean age, 19 years. Control: n = 623, mean age, 19 years	No difference in mean number of ANC visits attended (9 visits in both groups)
Robling; England; 2009 to 2010 [76]	Lancet; 2016	RCT: Structured home visits by family nurses from early pregnancy through 24 months’ postpartum	Primigravid adolescents aged ≤19 years; <25 weeks’ gestation; able to speak English	Intervention: n = 808, median age, 18 years. Control: n = 810, median age, 18 years	No difference in mean number of ANC visits attended (10 visits in both groups)
Sangalang; USA; 1991 to 1998 [73]	Health Soc Work; 2006	Retrospective cohort: Case management involving an average of 3 to 4 individual or group‐based contacts per month; including health information, counselling and involvement of family members	First‐time pregnant or parenting adolescents aged ≤18 years; comparison group matched on county of residence and age at delivery	Intervention: n = 1260, 69% aged 12 to 16 years. Comparison group: n = 1260, 67% aged 12 to 16 years	No difference in proportion attending ≥80% of expected prenatal visits (52% in intervention vs. 51% in comparison group)
Papers that reported that the approach examined significantly improved ANC attendance					
Allen; Australia; 2008 to 2012 [78]	Int J Nurs Stud; 2015	Retrospective cohort: Intervention (1): caseload midwifery (same midwife provides care through pregnancy, birth and postpartum); intervention (2): young women’s clinic in a community clinic setting; compared to control (standard of care)	Women aged ≤21 years when giving birth to a singleton with no congenital abnormalities; only included women who had attended at least 2 scheduled antenatal appointments	Intervention (1): n = 627, median age, 19 years. Intervention (2): n = 306, median age, 19 years. Control: n = 1038, median age, 20 years	Both interventions associated with a reduced likelihood of attending <5 ANC visits (intervention (1): 7% attended <5 visits; intervention (2): 8%; control: 12%)
Ashby; USA; 2007 to 2013 [70]	Psychol Serv; 2019	Pre‐post intervention: Trauma‐informed care implemented into an obstetric and paediatric medical home offering integrated services in one location for pregnant and parenting adolescents up to 22 years of age	Pregnant patients receiving care through the medical home	Pre‐intervention: n = 429, median age, 18 years. Post‐intervention: n = 415, median age, 19 years	Number of prenatal visits increased after implementation (from median of 6 pre‐intervention to 9 post‐intervention)
Dyalchand; India; 2008 to 2011 [79]	J Biosoc Sci; 2020	Pre‐post study in intervention and comparison areas: CHWs conducted monthly surveillance of adolescent reproductive health needs, facilitated referrals to care and provided behaviour change counselling	Married adolescent girls aged <20 years who had a live birth during the study period	Post‐intervention: Intervention: n = 526. Control: n = 276. Age not reported	Full use of ANC (entry into ANC <12 weeks’ gestation, consumption of iron folic tablets, receipt of tetanus toxoid injections and attending ≥3 ANC visits) improved from 8% to 56% in intervention areas (vs. 7% to 24% in control)
Fleming; Canada; 2004 to 2010 [77]	J Obstet Gynaecol Can; 2012	Matched cohort: Adolescent‐friendly antepartum and postpartum care providing “one‐stop” services	Adolescents aged <20 years who received prenatal care in an outreach programme, matched by age, year of delivery and hospital birth volume to control adolescents	Intervention: n = 206, mean age, 18 years. Control n = 831, mean age, 18 years	Higher proportion of intervention adolescents accessed ANC during first trimester (77% in intervention vs. 64%) and attended prenatal classes (53% in intervention vs. 30%)
Flynn; USA; 2002 to 2003 [71]	Public Health Nurs; 2008	Cohort with comparison group: Home visits conducted by nurses and social workers; including information about community resources, assisting with selecting a prenatal care provider and transport to healthcare appointments	Intervention: Aged ≤18 years at entry into programme; comparison group: aged ≤19 years at delivery	Intervention: n = 83, mean age, 17 years. Comparison: n = 216, mean age, 18 years	Comparing outcomes at entry into programme and after three months: proportion who did not have a prenatal care provider decreased (from 9% to 0%); and proportion who did not keep appointments decreased (from 11% to 1%). Compared to comparison group, all adolescents in programme received 6+ ANC visits, compared to 22% of adolescents in comparison group
Mersal; Egypt; 2008 to 2009 [75]	East Mediterr Health J; 2013	RCT: 3 to 4 sessions of prenatal counselling	Primigravid women aged <20 years; in second trimester; had not participated in previous health promotion programme; and had no comorbidities	Intervention: n = 47, mean age, 16 years. Control: n = 46, mean age, 17 years	Intervention increased proportion of adolescents with adequate adherence to antenatal follow‐up (intervention: 14% before counselling to 95% after counselling; control: 21% to 16%)
Trotman; USA; 2008 to 2010 [74]	J Pediatr Adolesc Gynecol; 2015	Retrospective cohort: Compared three groups: (1) Centring Pregnancy vs. (2) single‐ or (3) multi‐provider prenatal care	Pregnant adolescents aged 11 to 21 years	Centring Pregnancy: n = 50, mean age, 17 years. Single‐provider: n = 50, mean age, 16 years. Multi‐provider: n = 50, mean age, 16 years	Adolescents in Centring Pregnancy group more likely to attend all prenatal care appointments (62%, vs. 52% in single‐provider and 41% in multi‐provider groups)

ANC, antenatal care; CHW, community health worker; RCT, randomized controlled trial.

Three studies examined adolescent‐focused services as an approach to improve adolescent outcomes [70, 77, 78]. This typically included multidisciplinary care provided by specifically trained providers and delivered within a young women’s clinic [78] or as part of an outreach programme [77]. In addition, a study based at a medical home that offered a range of services to pregnant adolescents within a single location in the United States examined the provision of trauma‐informed pregnancy care, including psychological and psychiatric services [70]. These studies reported that the interventions resulted in higher proportions of adolescents accessing ANC during the first trimester of pregnancy [77], an increase in the median number of antenatal visits attended [70] and decreased likelihood of attending <5 antenatal visits [78].

Continuity of care with the same provider was another common strategy [70, 74, 78]. In one study conducted in Australia, care was delivered by the same midwife through pregnancy, birth and postpartum, and women had 24‐hour telephone access to their midwife throughout; this approach decreased the likelihood that women would attend <5 ANC visits [78]. The other two studies conducted in the United States reported that continuity of care by the same provider increased the number of ANC visits attended [70, 74]. In one study, adolescents received care from the same clinician throughout pregnancy within an integrated service [70].

Three studies examined home visits, including one in the United States in which pregnant adolescents received two home visits per month until delivery, with planned receipt of at least six visits [71]. In this study, home visits were conducted by public health nurses and social workers for the purpose of assessing adolescents’ needs, supporting adolescents to attend antenatal visits, and providing referrals for additional services, and home visits were associated with an increased number of antenatal visits attended [71]. In the second study, conducted in England, the intervention consisted of up to 64 home visits from family nurses between early pregnancy and two years' postpartum. This intervention had no effect on attendance at ANC visits and was noted to be extremely costly [76]. The third study was conducted in India, and demonstrated that home visits by community health workers combined with referrals to care and behaviour change counselling increased the use of ANC services among married adolescent girls [79].

Finally, two studies examined group ANC for pregnant adolescents. A retrospective cohort study conducted in the United States reported that the proportion of women who attended all ANC visits was higher among those attending group ANC (62%) compared to those accessing single‐provider (52%) or multi‐provider care (41%) [74]. In contrast, a cluster‐randomized controlled trial in the United States demonstrated no association between group ANC and the number of ANC visits attended, although women attending group ANC visits had more favourable outcomes including fewer infants who were small for gestational age [72]. Of note, this study reported substantial challenges to attendance at group ANC, with adolescents attending half of the group visits on average and one in five attending no group visits [72].

### 3.7 Learning from strategies to improve retention among adolescents, regardless of HIV status

Taken together, we found mixed evidence for strategies to improve ANC attendance among adolescents. Interventions shown to be effective included providing multidisciplinary adolescent‐focused services, often provided at the same location; continuity of care with the same provider; and home visits (with two of three studies demonstrating that the intervention was effective); two studies examined group ANC for pregnant adolescents, with mixed results and notable challenges in attendance. Alongside the results of the primary search, our review of interventions among adolescents regardless of HIV status also failed to yield clear insights into evidence‐based strategies that could inform efforts to address the needs of pregnant adolescents and young WLHIV. As noted above, a major difference between the results of this search and the primary search is the setting in which studies were conducted. All studies identified as part of the primary search were conducted in sub‐Saharan African countries, while most studies identified for the secondary search of general adolescent populations were conducted in high‐income countries. The approaches found to be effective in these high‐income settings may not be applicable to low‐resource settings with a high HIV burden. Many of the approaches identified were resource‐intensive, including one that provided up to 64 home visits to women during pregnancy and the postpartum period [76], potentially limiting their feasibility in resource‐limited settings. Similarly, continuity of care with the same provider may not be feasible in overburdened PMTCT programmes in resource‐limited settings.

### 3.8 Limitations

Several limitations of this review should be noted. First, it was beyond the scope of this work to contact authors and request that they provide age‐disaggregated data. In addition, we did not assess the risk of bias in the studies included. The potential for confounding in non‐randomized studies is a major concern, and the potential for publication bias cannot be excluded. We were restricted in our ability to report details of the studies included, in particular the behavioural theories in which they were grounded, due to this information not being routinely presented in papers. Finally, critiquing the design and content of interventions was beyond the scope of this review.

## 4 Conclusions

Pregnant adolescents and young WLHIV are a vulnerable population with multiple complex needs, and prioritizing this group for intensive retention interventions could improve outcomes for both mothers and infants [14]. However, our review highlights the lack of evidence‐based approaches to improve retention in care in this population, with clear implications for future research. In particular, this review highlights the lack of reporting of age‐disaggregated results in studies examining retention interventions for pregnant WLHIV. Previous reviews have similarly highlighted this issue in studies examining approaches to improve retention among non‐pregnant adolescents and adults [25]. To address this issue, journal reviewers and editors could consider routinely requesting age‐disaggregated analyses of intervention effects in published manuscripts. Furthermore, there is a critical need for studies to include adequate numbers of adolescents and young women in study samples to allow for age‐disaggregated analyses and to support research in the area of intervention development and evaluation for adolescent and young pregnant WLHIV specifically. Given the continued high incidence of both HIV and pregnancy among adolescents and young women in sub‐Saharan Africa, prioritizing the development and evaluation of approaches to improve retention in this population could help improve both maternal and child health.

## Competing interest

The authors declare no conflict of interest.

## Authors’ contributions

EJA, CAT, BN, AL and SM conceptualized and designed the review. KBrittain conducted the initial searches, screened all abstracts, and conducted the data abstraction with help from KB. KBrittain drafted the manuscript. EJA, CAT, BN, BO, JL, AL and SM critically reviewed and revised the manuscript. All authors read and approved the final manuscript.

## Supporting information

**Table S1**. Search strategies for PubMed to identify studies examining approaches to improve retention in antenatal and/or postpartum care among adolescents and young women living with HIV
