# Supplementary material for: Improving retention in antenatal and postnatal care: a systematic review of evidence to inform strategies for adolescents and young women living with HIV
Source: J Int AIDS Soc. 2021 Aug 27;24(8):e25770. doi: 10.1002/jia2.25770 (PMC8395389; doi:10.1002/jia2.25770)
Supplement: Supplementary file 1 — Table S1. Search strategies for PubMed to identify studies examining approaches to improve retention in antenatal and/or postpartum care among adolescents and young women living with HIV [file JIA2-24-e25770-s001.docx]

| **Supplementary Table 1.** Search strategies for PubMed to identify studies examining approaches to improve retention in antenatal and/or postpartum care among adolescents and young women living with HIV | |
| --- | --- |
| *Primary search: approaches to improve retention among adolescents and young women living with HIV* | |
| 1. | antenatal OR breastfeeding OR “breast feeding” OR maternal OR maternity OR mother OR mothers OR perinatal OR peri-natal OR postnatal OR post-natal OR postpartum OR post-partum OR pregnancy OR pregnant |
| 2. | HIV OR “human immunodeficiency virus” OR “human immune-deficiency virus” |
| 3. | antiretroviral OR anti-retroviral OR ARV OR ART OR “prevention of mother to child transmission” OR "prevention of mother-to-child transmission" OR PMTCT |
| 4. | attend* OR adher* OR attrition OR complian* OR complied OR comply OR default* OR disengag* OR engag* OR “loss to follow up” OR “loss to follow-up” OR “loss to followup” OR “lost to follow up” OR “lost to follow-up” OR “lost to followup” OR LTFU OR “loss to care” OR “lost to care” OR “loss to program” OR “loss to programme” OR “lost to program” OR nonadher* OR non-adher* OR noncomplian* OR non-complian* OR retain* OR retention |
| 5. | enhanc* OR decreas* OR improv* OR increas* OR optimis* OR optimiz* |
| 6. | approach OR approaches OR evaluation OR evaluations OR initiative OR initiatives OR intervention OR interventions OR model OR models OR package OR packages OR pilot OR pilots OR program OR programs OR programme OR programmes OR project OR projects OR strategy OR strategies OR trial OR trials |
| 7. | #1 AND #2 AND #3 AND #4 AND #5 AND #6 |
|  |  |
| *Secondary search: approaches to improve attendance at antenatal care and/or facility delivery among pregnant adolescents, regardless of HIV status* | |
| 1. | “antenatal care” OR “ante natal care” OR “antenatal healthcare” OR “antenatal health care” OR “antenatal health service” OR “antenatal health services” OR “antenatal service” OR “antenatal services” OR “perinatal care” OR “peri natal care” OR “perinatal healthcare” OR “perinatal health care” OR “perinatal health service” OR “perinatal health services” OR “perinatal service” OR “perinatal services” OR “prenatal care” OR “pre natal care” OR “prenatal healthcare” OR “prenatal health care” OR “prenatal health service” OR “prenatal health services” OR “prenatal service” OR “prenatal services” OR “maternal care” OR “maternal healthcare” OR “maternal health care” OR “maternal health service” OR “maternal health services” OR “maternal service” OR “maternal services” |
| 2. | attend* OR coverage OR uptake OR use OR usage OR utilis* OR utiliz* |
| 3. | #1 AND #2 |
| 4. | “facility delivery” OR “facility deliveries” OR “facility-based delivery” OR “facility-based deliveries” OR “facility based delivery” OR “facility based deliveries” OR “facility birth” OR “facility births” OR “facility-based birth” OR “facility-based births” OR “facility based birth” OR “facility based births” OR “clinic delivery” OR “clinic deliveries” OR “clinic birth” OR “clinic births” OR “hospital delivery” OR “hospital deliveries” OR “hospital-based delivery” OR “hospital-based deliveries” OR “hospital based delivery” OR “hospital based deliveries” OR “hospital birth” OR “hospital births” OR “hospital-based birth” OR “hospital-based births” OR “hospital based birth” OR “hospital based births” OR “hospital childbirth” OR “hospital childbirths” OR “institutional birth” OR “institutional births” OR “institutional childbirth” OR “institutional childbirths” OR “institutional delivery” OR “institutional deliveries” |
| 5. | #3 OR #4 |
| 6. | adolescent OR adolescents OR adolescence OR teenage OR teenager OR teenagers OR youth |
| 7. | enhanc* OR improv* OR increas* OR optimis* OR optimiz* |
| 8. | approach OR approaches OR evaluation OR evaluations OR initiative OR initiatives OR intervention OR interventions OR model OR models OR package OR packages OR pilot OR pilots OR program OR programs OR programme OR programmes OR project OR projects OR strategy OR strategies OR trial OR trials |
| 9. | 5# AND #6 AND #7 AND #8 |
